# Building and Understanding the Minimal Self

**DOI:** 10.3389/fpsyg.2021.716982

**Published:** 2021-11-26

**Authors:** Valentin Forch, Fred H. Hamker

**Affiliations:** Department of Computer Science, Chemnitz University of Technology, Chemnitz, Germany

**Keywords:** minimal self, mechanistic models, cognitive robotics, sense of agency, sense of ownership

## Abstract

Within the methodologically diverse interdisciplinary research on the minimal self, we identify two movements with seemingly disparate research agendas – cognitive science and cognitive (developmental) robotics. Cognitive science, on the one hand, devises rather abstract models which can predict and explain human experimental data related to the minimal self. Incorporating the established models of cognitive science and ideas from artificial intelligence, cognitive robotics, on the other hand, aims to build embodied learning machines capable of developing a self “from scratch” similar to human infants. The epistemic promise of the latter approach is that, at some point, robotic models can serve as a testbed for directly investigating the mechanisms that lead to the emergence of the minimal self. While both approaches can be productive for creating causal mechanistic models of the minimal self, we argue that building *a* minimal self is different from understanding *the human* minimal self. Thus, one should be cautious when drawing conclusions about the human minimal self based on robotic model implementations and vice versa. We further point out that incorporating constraints arising from different levels of analysis will be crucial for creating models that can predict, generate, and causally explain behavior in the real world.

## Introduction

The minimal self describes the immediate, pre-reflective experience of selfhood derived from sensory information (Gallagher, 2000; Blanke and Metzinger, 2009). Conceptually, it has been subdivided into the sense of agency (SoA, “I produced an outcome with my voluntary action.”) and the sense of ownership (SoO, “This body part/mental state belongs to me”. Haggard, 2017; Braun et al., 2018). In the wake of experimental paradigms that added implicit measures to the verbally reported experience of SoA (Haggard et al., 2002) and SoO (Botvinick and Cohen, 1998), both concepts have received considerable attention in the behavioral, cognitive, and neurosciences (David et al., 2008; Blanke et al., 2015; Haggard, 2017; Noel et al., 2018). Currently, the field offers a wealth of empirical findings on the antecedents of and relationships among the implicit and explicit behavioral measures of minimal selfhood as well as related neurophysiological measures (see Blanke et al., 2015; Braun et al., 2018; Noel et al., 2018 for reviews).

These advances in the human domain have been paralleled by a growing interest in the different aspects of the minimal self among roboticists and AI researchers who reason that equipping machines with a self-representation similar to humans will ultimately increase their performance and robustness in real-world settings (e.g., Hoffmann et al., 2010; Legaspi et al., 2019; Hafner et al., 2020). Collaborative efforts of robotics and psychology have been spearheaded by cognitive robotics and further advanced by developmental robotics, which strives for the implementation of a quasi-human developmental scheme for robots (Asada et al., 2009). More specifically, an agentive model embodied by a robot undergoing a developmental phase like human infants could enable direct investigations into the mechanisms that lead to the emergence of a minimal self (Hafner et al., 2020) and thus could be used to test different theories regarding the minimal self.

Current theoretical accounts on the minimal self may be broadly categorized into (a) informal models, including box-and-arrow models and verbal formulations of laws and constraints for the emergence of SoO and SoA (e.g., Synofzik et al., 2008; Tsakiris, 2010; Blanke et al., 2015; Haggard, 2017), (b) Bayesian accounts, according to which the perception of SoO and SoA is governed by statistically optimal information integration, as a main function of the brain is to optimally estimate the state of the world (e.g., Samad et al., 2015; Legaspi and Toyoizumi, 2019), and (c) accounts based on the free energy principle (FEP), which also lends itself to the interpretation of the self as the result of a continuous process of optimizing one’s world model (e.g., Limanowski and Blankenburg, 2013; Apps and Tsakiris, 2014; Seth and Friston, 2016).

Much of this theorizing regarding the minimal self is non-mechanistic in the sense that it either focuses on the computational level of cognition (Marr, 1982), which is about describing goals rather than the underlying mechanisms, or does not specify how relevant brain functions are carried out by specific parts of the brain. In more statistical terms, this could be expressed as defining the objective function that needs to be optimized by an agent without specifying the algorithms *the agent* employs to do the optimization. However, if one is interested in building mechanistic models – ones that can causally explain psychological phenomena – it is crucial to account for the algorithmic/representational and implementational levels (Marr, 1982), which describe how and by which parts the goals specified on the computational level are achieved (Piccinini and Craver, 2011; Love, 2015; Kriegeskorte and Douglas, 2018).^1^

The problem of neglecting mechanistic details becomes acute when the use of robotic platforms necessitates model implementation. If a model is underconstrained on the representational and implementational level, researchers will be forced to choose between many algorithms which can achieve the specified computational goal(s; cf. Anderson, 1978). In turn, this is likely to produce a significant deviation of the model from human behavior as not all algorithms for achieving a given computational goal perform equally under non-optimal conditions (e.g., time pressure, insufficient memory capacity, and internal noise) which are characteristic for the real-world settings humans operate in Wang (2019). Moreover, without specifying further constraints, human information integration appears to be non-optimal for many tasks (Rahnev and Denison, 2018; Lieder and Griffiths, 2020). The question of how to reconcile these idiosyncrasies with theories of optimal information integration has sparked an ongoing debate (also see Bowers and Davis, 2012; Griffiths et al., 2012; Love, 2015). In a similar vein, one should consider the context and complexity of the behavior to be modeled (Craver, 2006; Krakauer et al., 2017) – superficial phenomenal descriptions will likely lead to over-simplistic models.

In sum, whatever aspects of the minimal self (or any target system), a model can represent should depend on three factors: (a) the model’s objective function or goal (e.g., optimal prediction of the environment and solving a set of tasks), (b) the algorithmic implementation it employs for achieving its goals, and (c) the conditions under which it operates or inputs it receives. We assume that only if all three factors align, the model can serve as a mechanistic explanation. Conversely, if mechanistic details are not specified and phenomenal similarities between humans and robots are superficial, drawing conclusions from model implementations to humans (and vice versa) would be ill-advised.

Thus, the present contribution aims at highlighting the need for deeper integration of insights from the behavioral, cognitive, and neurosciences if one’s goal is a better understanding of the human minimal self. Of course, the interactive approach of robotics and ideas from artificial intelligence benefit cognitive neuroscience (Marblestone et al., 2016; Hoffmann and Pfeifer, 2018). We contend, however, that only models of the human minimal self which are phenomenologically rich and specify mechanistic details can be meaningfully tested through robotic model implementations. In the remainder, we will go into more detail regarding (a) the role of causal mechanistic models in cognitive neuroscience, (b) the mechanistic depth of different models of aspects of the minimal self, and (c) the current state of cognitive and developmental robotics implementations of such models.

## Causal Mechanistic Models in Cognitive Neuroscience

Understanding a phenomenon requires being able to explain how said phenomenon comes about (or fails to do so) under certain circumstances. Such causal explanations need to specify the mechanism producing said phenomenon (Craver, 2006). A mechanism is defined as being composed of parts whose organized activity produces a phenomenon from certain starting conditions (Machamer et al., 2000; Craver, 2006). Crucially, there needs to be a clear relation between parts and processes (Hommel, 2020) and the assumed parts of the mechanism need to be measurable and open to intervention to make the causal model testable (Craver, 2006).

The notion of causal mechanistic models does not imply reductionism (Nicholson, 2012), that is, that human behavior can be explained satisfactorily in the language of neuroscience, molecular biology, or particle physics alone. Rather, it is open to multilevel explanations (Kaplan and Craver, 2011). Crucially, this also requires a thorough description of the phenomenon to be explained and a distinction between standard and non-standard (e.g., lab) conditions (Craver, 2006). If the conditions under which a phenomenon is observed and described are non-representative of the real world, a model trying to explain it will likely not generalize well to real-world scenarios. Models in (computational) neuroscience have been criticized for being too reductionist, focusing on biological mechanisms that cannot be related to meaningful behavior (Krakauer et al., 2017).

Descriptive models, on the other hand, act as a compact summary of a phenomenon (Kaplan and Craver, 2011). They enable predictions about the phenomenon, without specifying the underlying mechanism. This type of model is widespread in psychology and cognitive neuroscience (Kaplan and Craver, 2011; Hommel, 2020; Litwin and Miłkowski, 2020) and can be derived from general assumptions about brain function (e.g., “the brain optimizes an internal world model”) or empirical observations (e.g., the rubber hand illusion, brain imaging data). A descriptive model can still serve as a starting point for building a causal model if it is possible to relate parts of the model to parts of a causal mechanism (Kaplan and Craver, 2011; Piccinini and Craver, 2011). Moreover, in the face of physiological and behavioral complexity, the notion of a truly mechanistic model appears somewhat idealized and may be only approached gradually, making descriptive models a reasonable starting point.

## Mechanistic Depth of Models of the Minimal Self

Starting with informal descriptive models of the minimal self, we will consider the work by Tsakiris (2010) (see also Wegner and Wheatley, 1999; Frith et al., 2000; Synofzik et al., 2008; Chambon et al., 2014; Blanke et al., 2015). This model is concerned with explaining the SoO over body parts or objects. It proposes a tiered comparison between the features of candidate objects for experiencing ownership and the current state of an internal body model (i.e., comparison of visual appearance, posture, and sensory stimulation – in this order). Tsakiris (2010) also points toward evidence of certain brain areas being responsible for this comparison. While the model provides an algorithm in the sense that it specifies the order in which certain information is compared, it includes no constraints on the algorithms for making the comparisons or how they could be implemented by the brain. It also does not specify how the internal model of the body is represented.

**Figure 1:**
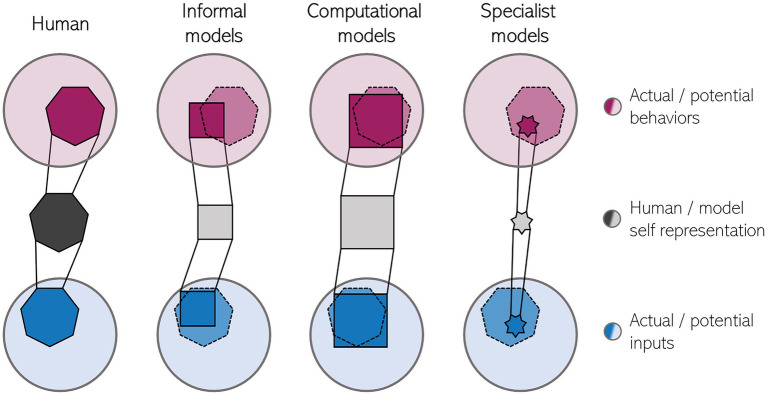
Schematic of the representational power of models for the human minimal self. The human minimal self representation (dark grey, left) is based on a relevant subset of all self-related information (blue) while also underlying a subset of all possible self-related behaviors (purple, e.g., experiencing a piece of furniture as belonging to oneself). Currently, models of the self (right) are too narrow in the sense that they consider only a subset of potential inputs and in practice can generate only a small subset of human behaviors and/or too general in the sense that they make too unspecific predictions regarding self-related phenomena, violating the bounds of the human self (dotted line). Note, that the relative number of constraints underlying each self-representation is reflected by the number of sides of the respective shapes.

Although the model makes testable predictions, it is clearly not mechanistic to the degree that it would permit a straightforward robotic implementation without additional assumptions. The same holds for other informal models which specify what kind of information is processed, but which do not provide the actual metric used for making comparisons or the processes underlying the formation of representations. Figure 1 tries to make a graphical comparison between the human self-representation and models of the human self. Informal models typically account for relatively broad phenomena like the SoO. Thus, they cover a large part of the human “self-space” (observable self-related behaviors and self-related information relevant for constructing the internal self-representation). However, as they are only loosely constrained by theoretical assumptions and do not make quantifiable predictions, these models would likely conform with behavior that is outside the human repertoire.

Bayesian models (e.g., Samad et al., 2015; Legaspi and Toyoizumi, 2019) frame the perception of SoA and SoO as the posterior probability for perceiving objects or actions as belonging to or being caused by oneself given sensory input and prior beliefs. These models can be very useful for untangling what information is relevant for a certain task or percept (e.g., Legaspi and Toyoizumi, 2019) but usually make no commitments to the algorithms employed by the brain (Griffiths et al., 2012; Love, 2015). Neurocomputational models for approximating Bayesian inference (e.g., Pouget et al., 2000) try to build a bridge between computational goals and concrete implementations (cf. Love, 2015) and have been shown to fit the response characteristics of biological neurons (Avillac et al., 2005).

While neurocomputational models for multisensory integration – which is thought to be central for the SoO – are abundant (see Ursino et al., 2014; Blanke et al., 2015 for reviews), there are still explanatory gaps: (a) many of these models feature no learning mechanism (e.g., Deneve et al., 2001) or use learning techniques that cannot be brought into correspondence with parts and processes of the brain (i.e., the use of machine learning techniques, Makin et al., 2013), (b) many models are based on physiological data from midbrain structures (e.g., Cuppini et al., 2012; Oess et al., 2020), where the empirical link between these structures and the perception of SoO is not clear, and (c) the neurophysiological constraints incorporated into these models so far have not been demonstrated to give rise to more specific predictions on the behavioral level.

The latter point is important because traditional Bayesian models and thus their neurocomputational counterparts often only apply to human behavior in idealized situations (Love, 2015; Rahnev and Denison, 2018). Research from other domains, however, has shown that taking additional constraints on the representational level (e.g., efficient coding; Wei and Stocker, 2015) or implementational level (e.g., internal noise; Tsetsos et al., 2016) into account can greatly benefit modeling “non-optimal” human behavior in real-world settings (also see Lieder and Griffiths, 2020 for a review). These examples show that by refining computational models with more low-level constraints instead of simply translating them into a neurocomputational framework, it is possible to move closer to the human style of information processing – also an exciting opportunity for research on the self.

The FEP builds on the notion that human brains, like all living systems, can be thought of as “trying” to minimize their surprisal through representing an optimal world model and acting on it (Friston, 2010). At its core, the FEP is closely related to Bayesianism (Aitchison and Lengyel, 2017) but incorporates a (variable) host of additional assumptions (Gershman, 2019; Bruineberg et al., 2020), the most important arguably being the explicit representation of prediction errors at all stages of perception and action, termed predictive coding (PC, Rao and Ballard, 1999; see Aitchison and Lengyel, 2017 for PC schemes in other contexts). According to PC, predictions descend the cortical hierarchy where they suppress incoming bottom-up signals leading to the representation of prediction errors. These prediction errors, in turn, are propagated up the hierarchy to inform the update of higher-level representations. Ultimately, this leads to a dynamic equilibrium where prediction errors are minimized (Friston, 2010).

The FEP and PC have been rapidly adopted in the domains of interoception and the (minimal) self (e.g., Limanowski and Blankenburg, 2013; Apps and Tsakiris, 2014; Barrett and Simmons, 2015; Seth and Friston, 2016). Building on PC, Apps and Tsakiris (2014), for instance, explain illusions of ownership over extracorporeal objects like the rubber hand illusion as a process where prediction errors caused by incongruent sensory information are “explained away” by updating one’s high-level representations in such a way that best predicts said sensory information. However, the authors do not specify how the prediction errors are computed or how they are transformed into beliefs.

This gap may be closed by neurocomputational models of PC (Bastos et al., 2012). However, as neurophysiological evidence for PC is inconclusive (Seth and Friston, 2016; Aitchison and Lengyel, 2017), this vein of research requires further investigation (Keller and Mrsic-Flogel, 2018). Additionally, the same reservation as for Bayesian models applies – in our view, showing that an optimization scheme can be implemented through neural computation, while being necessary for a possible mechanistic explanation, is not sufficient as long as the more specific model does not capture relevant deviations from behavior predicted by computational constraints alone.

One such deviation yet unexplained by computational models may be the apparent dissociation of explicit and implicit measures of SoO in the rubber hand illusion under certain conditions (Holle et al., 2011; Rohde et al., 2011; Gallagher et al., 2021), which has been explained under the same framework of information integration (Apps and Tsakiris, 2014). Another example is the effect of action selection fluency on SoA (Chambon et al., 2014) which shows that the SoA can be diminished solely by hindering fluent action selection. This effect is independent of the predictability of the action outcome – the core tenet of comparator models of SoA (Frith et al., 2000) which strongly align with PC (cf. Aitchison and Lengyel, 2017). Coming back to Figure 1, we would then argue that, albeit being very broad in scope, computational models of the minimal self are only a first approximation of the information processing underlying the minimal self. Refining these models with new constraints will necessitate synergistic modeling and empirical work – behavioral scientists will have to further explore the limits of the malleability of the human minimal self and the relative importance of different kinds of information used for constructing it, thereby informing theorists who, in turn, should create models that make new, empirically testable predictions, thus entering an experiment-model development-prediction cycle of research. One concrete future direction might be considering multiple computational constraints which could even play different roles during development (cf. Marblestone et al., 2016). Besides prediction error reduction this could be, for instance, novelty, reward maximization, or computational efficiency.

## Minimal Self-Models in Cognitive and Developmental Robotics

Applying a theory or model in a complex environment either through simulation or the use of physical robots may speed up research efforts significantly by reducing the need for time-consuming human experiments and increasing the control and transparency of the subject. Unfortunately, reviewing robotic models related to the minimal self would be beyond the scope of this contribution (see Nguyen et al., 2021 for an excellent review). Instead, we want to point out two tendencies that may impair the epistemic power of robotic model implementations.

Compared to traditional cognitive and neuroscience models, robotic implementations have the advantage of receiving rather realistic input as robots can directly interact with the real world and register the consequences of their actions (Hoffmann and Pfeifer, 2018). Moreover, the use of embodied agents allows testing the impact of physiological features (i.e., body morphology) on learned representations. This increased fidelity of model inputs, however, makes implementations much more demanding. Thus, it is not surprising that robotic model implementations often rely on more scalable machine learning techniques instead of neurocomputational models (cf. Nguyen et al., 2021). This has the benefit of introducing powerful ideas like curiosity-driven learning (Oudeyer et al., 2007), but also contains the risk of deviating on the algorithmic level by choosing an algorithm that elegantly solves a given task while neglecting biological constraints. We assume this concern will bear greater importance when task complexity increases and experimental settings move closer toward the real world.

Second, as Krichmar (2012) noted, cognitive robotics models, in general, tend to be built to perform very specific tasks. This diminishes the ecological benefit of real-world inputs because it greatly reduces the possible robot-world interactions. Moreover, the use of narrow tasks holds the risk of over-engineering the model to the task (as, e.g., Hoffmann et al. (2021) note for robotic models of minimal self-awareness). Such specialist models will hardly generalize in novel situations. Covering the whole self-space (Figure 1) would then require a multitude of such models that need to be integrated somehow, which would be a daunting task (Clune, 2020). Moreover, testing a robotic implementation under quasi-lab conditions only for the behaviors which have been used to build and train the underlying model cannot be regarded as a critical test of a theory.

One promising approach, therefore, appears to be letting robots solve general tasks that necessitate real-world interactions without explicitly engineering the model to perform a specific behavior, like say, attenuating self-caused sensory input – which has been related to SoA (Schillaci et al., 2016; but see Kaiser and Schütz-Bosbach, 2018). In such a scenario, the robot should show some behavior because it is (a) possible and (b) beneficial for task success. One could then proceed by probing the conditions under which this behavior develops or is enacted. By comparing the model to human behavior under diverse conditions, one could simultaneously test the assumed mechanism and deepen the phenomenological description of the human repertoire. This method could even be generalized to the point where the agent is not designed by the researcher but by an (evolutionary) algorithm guided by task success and prior constraints (cf. Albantakis et al., 2014). However, such an approach might require going to the edge of what is currently computationally possible (cf. Clune, 2020).

## Discussion: Why Mechanistic Models?

So far, we have established that there is no complete mechanistic explanation of the minimal self yet – but why should mechanistic models be beneficial for further research on the minimal self? We see several benefits in striving for integrating evidence from different levels of description and thereby creating more mechanistic models of the minimal self: (a) It safeguards against overfitting to specific pieces of evidence, assumptions, or tasks, (b) it increases model comparability and the probability of model generalization, and (c) especially in clinical contexts, a causal understanding may help to find effective interventions for (self-)disorders and interfaces with other theories (e.g., Schroll and Hamker, 2016; Neumann et al., 2018). For brevity, we will only touch upon the first two points.

Anchoring a model in a narrow set of observations, assumptions, or tasks bears the risk of selectively including evidence that fits the model and tailoring the model to these data points (cf. Love, 2015). Because mechanistic models demand a multilevel view on a phenomenon, their implementation should counteract this risk. They should also increase model comparability as there can be no meaningful comparison of two models that make predictions for distinct variables or solve different tasks (Love, 2021). As the minimal self and its subcomponents are relevant in many contexts, their corresponding mechanistic models should also not be bound to a narrow task.

Furthermore, explicitly distinguishing between mechanistic and non-mechanistic models also helps when thinking about robots as models for the human minimal self. If we understand the self as a representation of contextually and ethologically relevant features of one’s physical body and intentional actions which is learned and continuously updated by the nervous system, we may ascribe a minimal (pre-reflective) self to very primitive creatures like ants. Ants have been shown to perform approximately optimal cue integration of vision and proprioception (Wystrach et al., 2015),^2^ act intentionally (Hunt et al., 2016), and learn (Dupuy et al., 2006). Admittedly being an exaggeration, this example should make clear that if we exclude higher-order cognition (as it is not pre-reflective), ignore individual representational capacities, behavioral complexity, and other conditions constraining sensory content, we run the risk of ascribing some phenomenology to systems vastly different from us.

Certainly, there is much potential in using embodied machines to advance investigations into the human minimal self. However, we would caution against thinking of both as being representative for one another as long as there is no agreement between all levels of description relevant for cognition and behavior. This should not imply that robot “brains” or other models need to be neuromorphic, but as the human brain is a product of the chaotic process of evolution, and given that there is no unique implementation of purely computational theories due to the complexity and dynamics of real-world settings (Whiteley and Sahani, 2012; Gershman, 2019), it appears unlikely that an algorithm that is only constrained by a single computational goal could fully capture human behavior and experience (cf. Marblestone et al., 2016; Kriegeskorte and Douglas, 2018; Lieder and Griffiths, 2020). In conclusion, incorporating constraints arising from different levels of analysis will be crucial for creating models able to predict, generate, and mechanistically explain behavior related to the minimal self in the real world.

## Data Availability Statement

The original contributions presented in the study are included in the article/supplementary material, further inquiries can be directed to the corresponding author.

## Author Contributions

VF and FH jointly developed the general idea discussed in the manuscript. VF wrote the manuscript and produced Figure 1. FH reviewed the final manuscript. All authors contributed to the article and approved the submitted version.

## Funding

This work was supported by the DFG priority program “The Active Self” HA2630/12-1.

## Conflict of Interest

The authors declare that the research was conducted in the absence of any commercial or financial relationships that could be construed as a potential conflict of interest.

## Publisher’s Note

All claims expressed in this article are solely those of the authors and do not necessarily represent those of their affiliated organizations, or those of the publisher, the editors and the reviewers. Any product that may be evaluated in this article, or claim that may be made by its manufacturer, is not guaranteed or endorsed by the publisher.

## Footnotes

^1^When talking about the implementational level, we do not exclusively refer to singular neurons or synapses. Groups of neurons or brain areas may also be related to a function. To be verifiable mechanistic parts, the states of such a physical system still need to be measurable and clearly attributable to the implementation of a concrete algorithm.

^2^The study of Wystrach et al. (2015) also provides an example of the importance of implementation constraints affecting behavior. Ants show “suboptimal” cue integration under some circumstances which could be explained by a memory restriction in their information processing.
